# Retrospective analysis of 52 patients with prolactinomas following endoscopic endonasal transsphenoidal surgery

**DOI:** 10.1097/MD.0000000000013198

**Published:** 2018-11-09

**Authors:** Yan-Long Han, Dong-Ming Chen, Cheng Zhang, Miao Pan, Xiao-Peng Yang, Yong-Gang Wu

**Affiliations:** a Department of Neurosurgery, People's Hospital of Xinjiang Uygur Autonomous Region, Urumqi; b Medical Graduate School of Shihezi University, Shihezi, Xinjiang, China.

**Keywords:** endoscopic endonasal transsphenoidal approach, neuro-endoscopy, prolactinoma, surgical effect

## Abstract

**Background:**

Prolactinomas affect patients’ quality of life and even endanger lives. The study aimed to investigate the effect of the endoscopic endonasal transsphenoidal approach (EETA) on 52 patients with prolactinomas.

**Methods:**

A total of 52 patients with prolactinomas who had previously undergone EETA in the People's Hospital of Xinjiang Uygur Autonomous Region between January 2013 and December 2017 were retrospectively analyzed. Factors affecting the extent of resection and postoperative remission rates were also investigated.

**Results:**

All the patients were pathologically diagnosed with prolactinomas. Compared with giant adenomas, the total removal rate of microadenomas and macroadenomas was significantly increased (*P* < .05). In addition, the total removal rate of patients with noninvasive prolactin adenomas was significantly higher than patients with invasive prolactinadenomas (*P* < .05). Furthermore, there were no significant differences in postoperative remission rates among patients with prolactin adenomas from different ethnic groups (*P* > .05). Also preoperative administration of bromocriptine and preoperative prolactin (PRL) levels did not significantly affect therapeutic outcomes postsurgery (*P* > .05). Postoperative menstruation was improved or normalized in 20 (38.5%) female patients, vision was improved or normalized in 15 (28.8%) patients, and headaches were improved or normalized in 22 (42.3%) patients. Sexual function was improved in 2 male patients following surgery. A total of 6 patients exhibited a recurrence following surgery. A number of patients suffered from postoperative complications, including transient diabetes insipidus in 5 (9.6%) patients and postoperative transient cerebrospinal fluid leakage in 2 (3.8%) patients.

**Conclusion:**

The results of this study demonstrated that tumor size, preoperative PRL levels, and invasion of adenomas represent independent factors that can affect the success of surgery. The results suggested that EETA represents a therapeutic strategy for the treatment of patients with prolactinoma with high remission rates and low complication rates. Therefore, EETA should be considered a primary treatment for patients with prolactinomas who are not responsive to treatment with medical therapy.

## 1 Introduction

Pituitary adenomas, one of the most common intracranial tumors, can be clinically classified into functional adenomas and nonfunctional adenomas. Prolactin (PRL)-secreting pituitary adenomas are the most common functional pituitary adenoma and account for ∼45% of all pituitary adenomas.^[1,2]^ In general, prolactinomas occur in females more frequently than males,^[3]^ and are diagnosed due to more obvious clinical manifestations, such as amenorrhea-lactation syndrome; whereas the clinical symptoms in men, such as sexual impotence and a decreased libido, are frequently missed. Macroprolactinomas (≥1 cm in diameter) are more common than microprolactinomas (<1 cm in diameter).

Prolactinomas are a common cause of reproductive and sexual dysfunction.^[4]^ For example, women with prolactinomas exhibit irregular menstruation and galactorrhea, whereas men often experience sexual impotence and decreased libido. In addition, many patients exhibiting nontypical clinical symptoms present unilateral or bilateral vision loss and may also suffer from visual defects and headaches. Furthermore, such patients exhibit increased serum prolactin compared with healthy patients, and magnetic resonance imaging (MRI) can be used to investigate the size and extent of the tumor.

The first-line therapy of patients with prolactinomas is treatment with dopamine agonists (DA), which can normalize PRL levels and reduce tumor volumes in ∼80% of patients.^[5,6]^ Patients who are medically nonresponsive or intolerant to drug therapy are subsequently obliged to cease drug treatment. Therefore, operative treatment has become the main therapeutic treatment for patients with prolactinomas. For decades, neurosurgery techniques regarding advances in the treatment of pituitary adenomas have rapidly developed. The majority of patients with pituitary adenomas undergo endoscopic endonasal transsphenoidal surgery, which has been suggested to be a highly effective therapeutic strategy, with a high remission rate and limited complications.^[7]^

The present study aimed to investigate the safety and efficacy of endoscopic endonasal therapy for the treatment of prolactinomas. The present study retrospectively analyzed patients with prolactinoma over a 5-year period who had previously undergone endoscopic endonasal transsphenoidal surgical intervention.

## 2 Materials and methods

### 2.1 Patient population

A total of 52 patients (38 females and 14 males; mean age of 37.69 ± 11.96 years old) with prolactinoma who had undergone endoscopic endonasal transsphenoidal surgery between January 2013 and December 2017 at the People's Hospital of Xinjiang Uygur Autonomous Region were retrospectively analyzed in the present study. A total of 23 patients were of Han nationality, 23 patients were of Uygur nationality, 5 patients were of Kazak nationality, and 1 patient was Russian (Table 1). The study gained the approval of the People's Hospital of Xinjiang Uygur Autonomous Region.

**Table 1:**
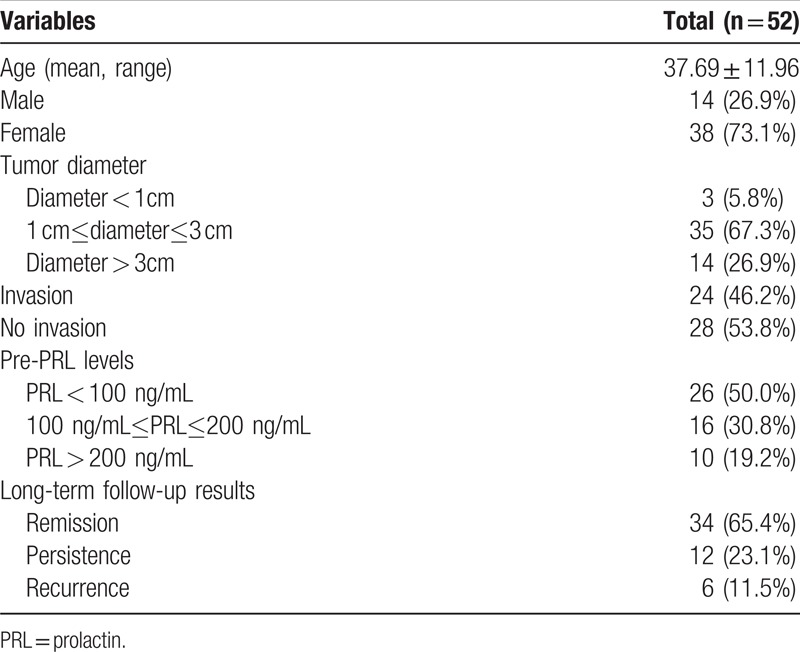
Patients and tumor characteristics.

In the present study, surgical therapy was performed on patients suffering from DA intolerance or DA resistance, visual field defects, pituitary hemorrhaging, or giant adenomas. Furthermore, patients who had chosen to undergo surgical intervention rather than chronic medical treatment with DAs were also investigated. In addition, PRL was classified as a failure to achieve normal levels and tumor size was classified as a failure to achieve tumor size reduction of ≥50% following maximal conventional doses of medication (7.5 mg/d of bromocriptine or 2.0 mg/wk of cabergoline), respectively.^[8]^

### 2.2 Clinical and physical symptoms

A total of 25 female patients exhibited irregular menstruation or amenorrhea, with or without galactorrhea. Two male patients exhibited sterility and decreased sexual function. A total of 18 patients exhibited unilateral or bilateral vision loss, with or without visual field defects. A total of 22 patients suffered from varying degrees of headaches, and 6 patients experienced recurrence postsurgery (Table 2).

**Table 2:**
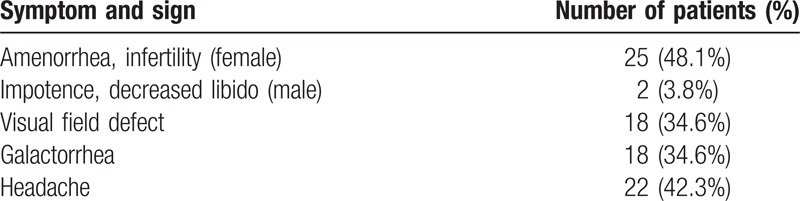
The clinical symptoms for 52 patients.

### 2.3 Determination of endocrine levels

Endocrine levels of all patients prior to surgery and postsurgery were determined at the People's Hospital of Xinjiang Uygur Autonomous Region. Levels of serum growth hormone (GH), insulin-like growth factor-1, GH following oral glucose tolerance test, prolactin, adrenocorticotrophic hormone, cortisol, thyreoid-stimulating hormone, free thyroxine, luteinizing hormone, follicle stimulating hormone, testosterone, and estradiol were also determined. Preoperative PRL levels were 25.15 ∼>300 ng/mL, the laboratory results were >300 ng/mL in our hospital, and there was no exact number when PRL levels were more than 300. Following tissue removal, all tumors were subjected to immunohistochemical analysis. According to preoperative PRL levels, the patients were classified into 3 categories: PRL < 100, 100 ≤ PRL ≤ 200, and PRL > 200 (Table 1).

### 2.4 Neuroimaging examination

**Figure 1:**
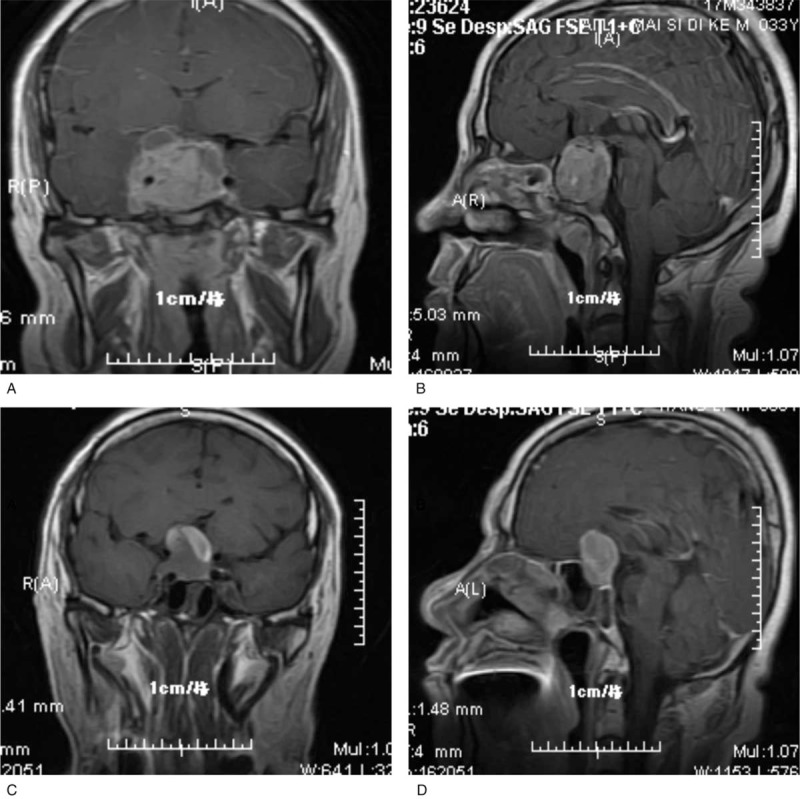
Preoperative MR. MR = magnetic resonance.

**Figure 2:**
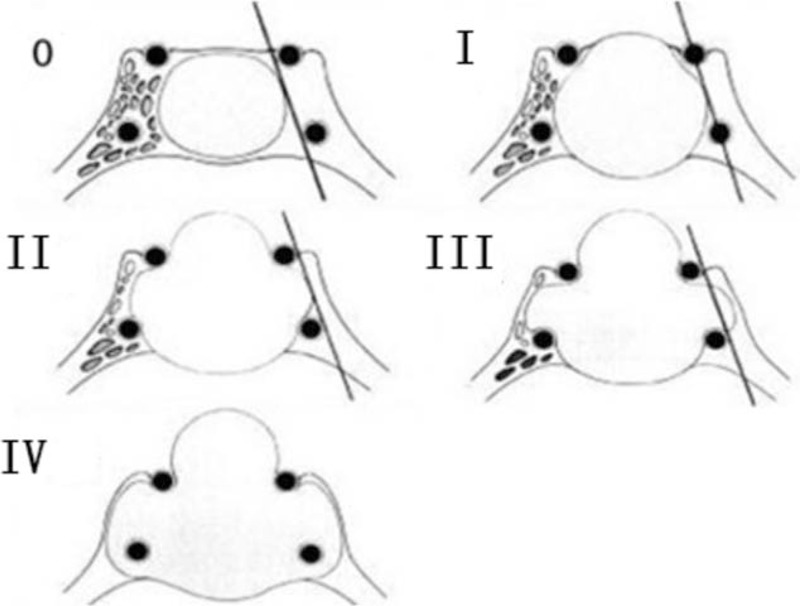
The Knosp–Steiner classification.

**Figure 3:**
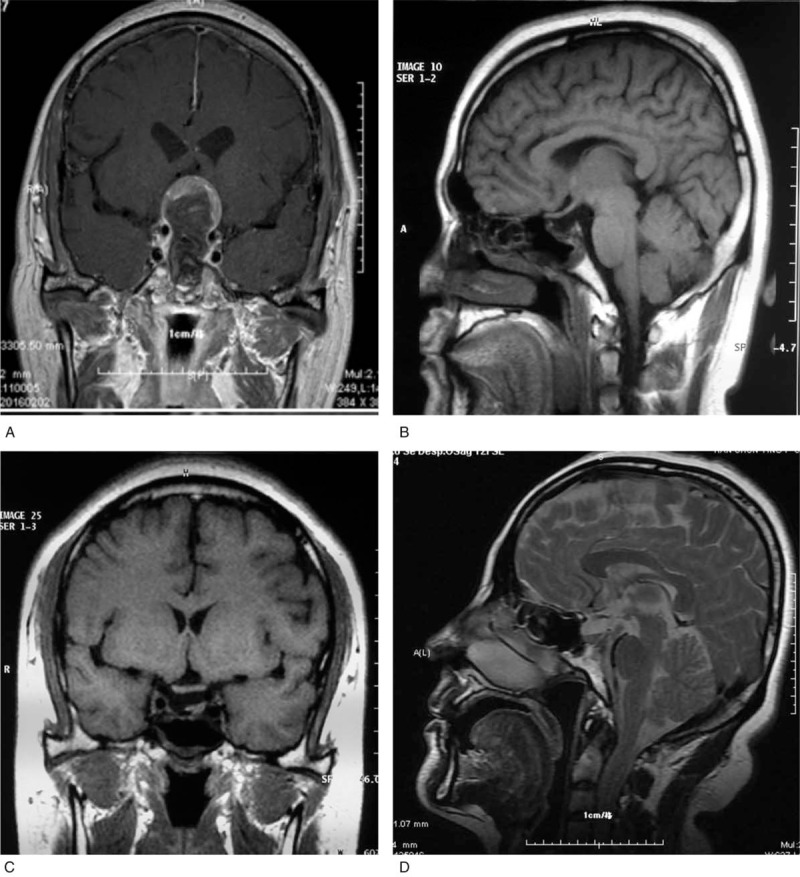
Postoperative MR. MR = magnetic resonance.

All patients were subjected to MRI examination to investigate tumors prior to surgery (Fig. 1). Anatomical alterations in the sellar floor, nasal cavity, and paranasal sinuses were investigated in all patients via facial sinus computed tomography (CT) scans. According to maximum tumor diameter, the size of tumors was classified into 3 categories: Microadenomas (<1 cm); macroadenomas (1–3 cm); and giant adenomas (>3 cm). Cavernous sinus invasion was analyzed according to the Knosp–Steiner classification^[9]^ (Fig. 2 and Table 3). Follow-up MRI examinations were performed on 1 day, 3, 6, and 12 month time intervals following surgery (Fig. 3).

**Table 3:**
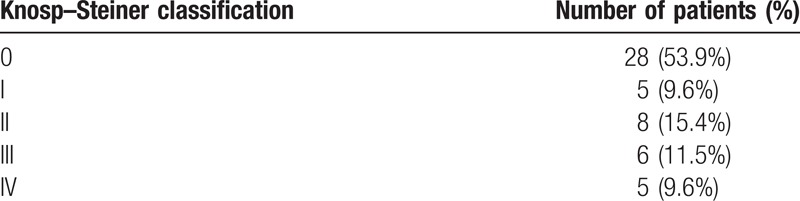
The Knosp-Steiner classification for 52 patients.

### 2.5 Surgical approach

**Figure 4:**
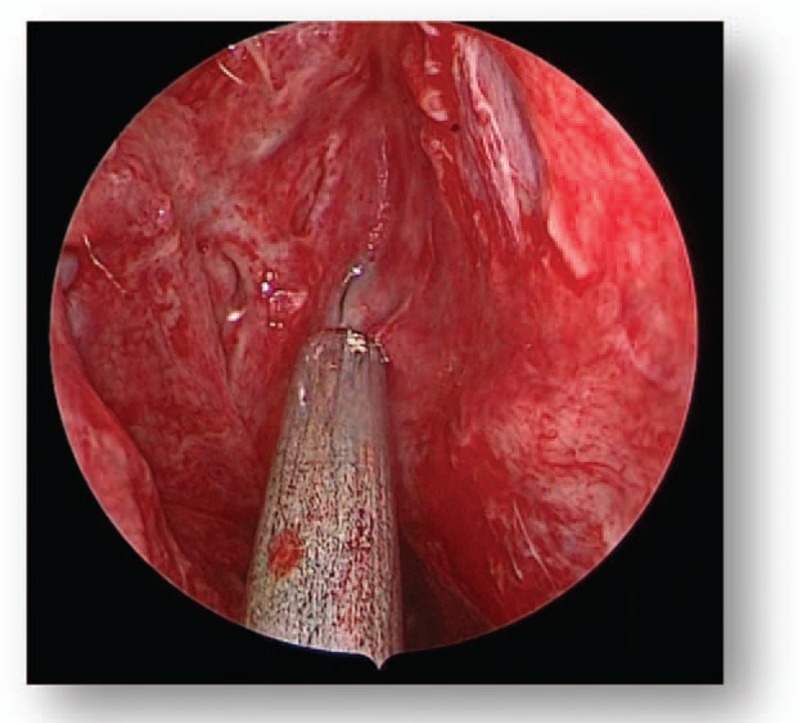
Preoperative MRI (A). MRI = magnetic resonance imaging.

**Figure 5:**
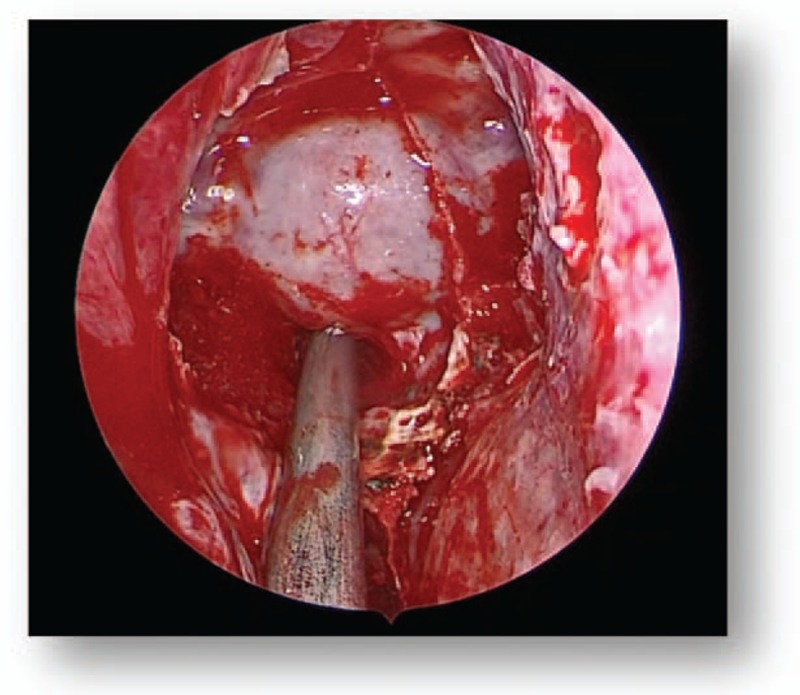
Preoperative MRI (B). MRI = magnetic resonance imaging.

**Figure 6:**
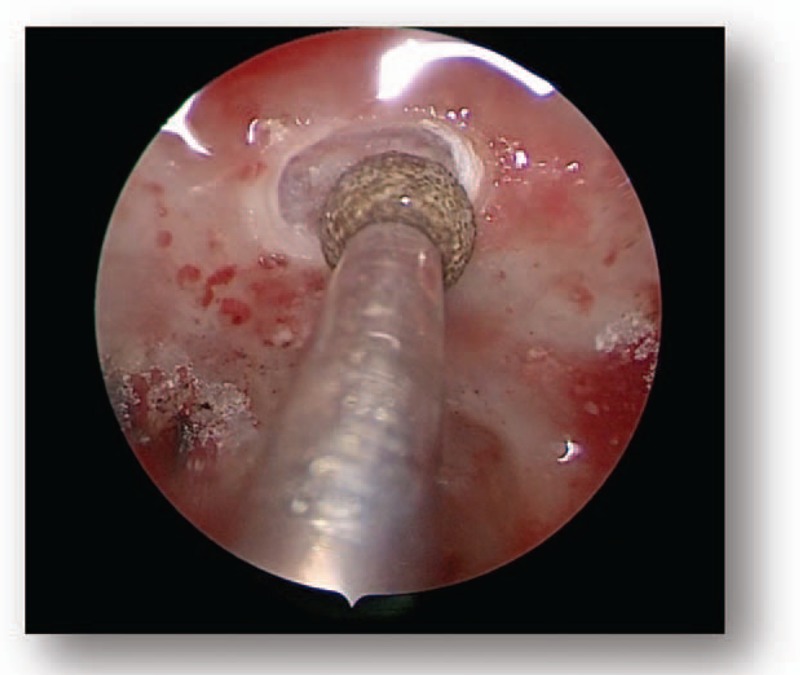
Preoperative MRI (C). MRI = magnetic resonance imaging.

**Figure 7:**
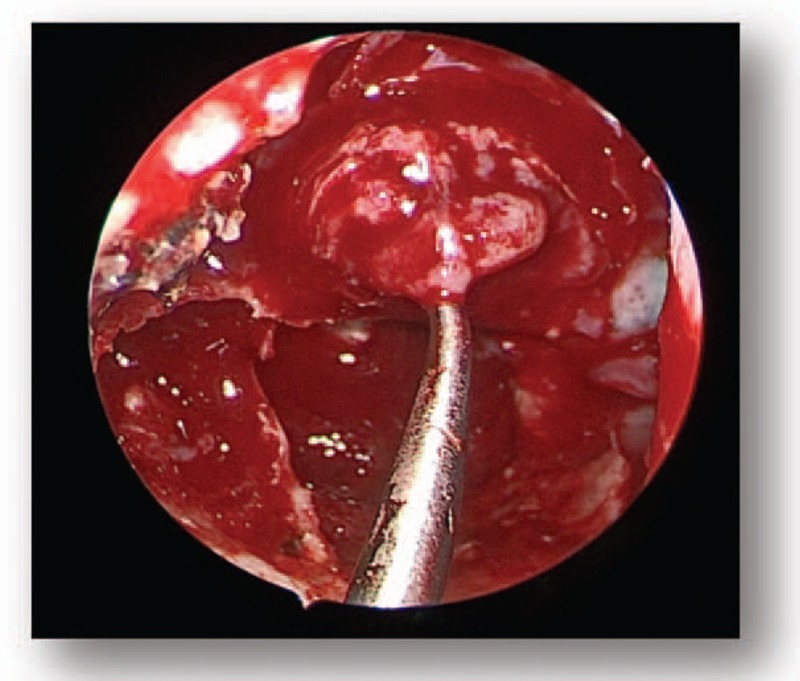
Preoperative MRI (D). MRI = magnetic resonance imaging.

All patients were subjected to endoscopic endonasal transsphenoidal surgery. Following general anesthesia, the patient was placed in a supine position and the head was elevated at a 15° angle and rotated toward the surgeon at a 25° angle. The operator was positioned on the right side of the patient. One nostril was chosen following the results of the CT and MRI images of the head; however, the right nostril was the most frequently chosen. The operation was performed between the nasal septum and the middle turbinate via the guidance of an endoscope angled at a 30° angle. Numerous cotton patties were inserted between the middle turbinate and the nasal septum to gradually widen the space. The nasal mucosa was separated and the mucosa surrounding the sphenoid ostium was coagulated via electrocoagulation to expose the sphenoid ostium (Fig. 4). Following this, a 1.5 to 2.0 cm diameter incision from the inferior margin of the middle turbinate to the sphenoidal sinus ostium was performed using a high-speed drill. Following the opening of the sphenoid sinus, numerous small septa in the sphenoid sinus were observed and trimmed using a high-speed drill or forceps, and the tuberculum sella, the clival indentation, and cavernous sinus were subsequently visible in the vertical and transverse dimensions (Fig. 5). The sellar floor was trimmed using a high-speed drill to create a 1 to 1.5 cm diameter incision (Fig. 6). The dura was subsequently coagulated with the bipolar coagulator, a transfixion pin was inserted into the sellar floor for demonstrating to be safely secured, and the dura was then incised into an “X” form using a sharp knife. Following this, the tumor tissue was exposed, scraped using a curet, and subsequently removed via a suction cannula (Fig. 7). The tumor resection cavity in the saddle area was appropriately filled with gelatin sponge, and the sellar floor was reconstructed using artificial dura to prevent postoperative cerebrospinal fluid leakage. Finally, the nasal mucosa was reconstructed and the nasal cavity was filled using oil gauze.

### 2.6 Tumor control

The criteria of successful treatment were total tumor removal and normalization of postoperative PRL levels. Whether the tumor had been completely removed was determined via the surgeon's intraoperative vision and subsequent MRI analysis 3 months postoperation. Subtotal resection was classified as tumor removal of between 80% and 100%; and partial resection was classified as tumor removal of <80%. Furthermore, a postoperative serum prolactin level of <20 ng/mL^[10]^ was considered to represent successful treatment.

### 2.7 Postoperative follow-up

Follow-up appointments were held 3 to 24 months postsurgery (mean 13.5 months). During follow-ups, data regarding clinical symptoms, serum PRL levels, and MRI scans were collected. If clinical symptoms had disappeared and serum PRL levels were considered healthy, patients were classified as “in remission”. Furthermore, patients exhibiting persistently enhanced serum PRL levels in the presence or absence of radiological evidence were defined as having persistent prolactinomas. In addition, if plasma PRL levels increased again in the presence or absence of radiological evidence following previous remission, patients were defined as having recurrent prolactinomas.

### 2.8 Statistical analysis

Statistical analysis was performed using SPSS software (v.24.0; IBM SPSS, Armonk, NY). Categorical variables were analyzed using the *χ*^2^ test. Multivariate analysis of categorical variables was analyzed using logistic regression analysis. *P* < .05 was considered to represent a statistically significant difference.

## 3 Results

### 3.1 General characteristics

The characteristics of 52 patients with prolactinomas are presented in Table 1. According to MRI analysis, 3 patients (5.8%) had microadenomas (<1 cm), 35 patients (67.3%) had macroadenomas (1–3 cm), and 14 patients (26.9%) had giant adenomas (>3 cm). In addition, 24 patients (46.2%) were demonstrated to have invasive prolactinomas via MRI analysis, which were subsequently confirmed during surgery. The degree of tumor expansion was revealed using Knosp–Steiner classification and is presented in Table 3. Furthermore, the criteria of Knosp–Steiner Classification are presented in Fig. 2.

### 3.2 Extent of resection

Among the 52 patients, complete tumor removal was demonstrated in 32 patients (61.5%) and subtotal resection was demonstrated in 20 patients (38.5%). The results revealed that the total resection rate of the 3 patients with microadenomas was 100%, the total resection rate of the 35 patients with macroadenomas was 68.6%, and the total resection rate of the 14 patients with giant adenoma was 28.6%. The association between surgical resection and tumor size is presented in Table 4. The results revealed that there were 24 patients with invasive prolactin adenomas (29.2%) that exhibited a total removal rate postsurgery and 28 patients with noninvasive prolactin adenomas (89.3%) exhibited a total removal rate postsurgery. The association between invasive and surgical resection is presented in Table 5.

**Table 4:**
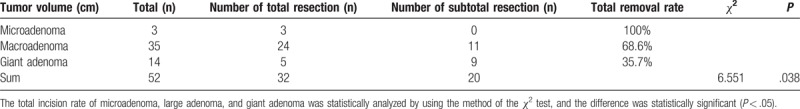
The relationship between surgical resection and tumor size.

**Table 5:**
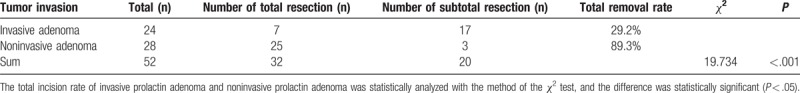
The relationship between invasive and surgical resection.

### 3.3 Clinical and endocrine outcomes

The clinical manifestations of 52 patients are presented in Table 3. In the female patients, 25 patients exhibited irregular menstruation or amenorrhea, with or without galactorrhea. Following EETA, 20 female patients exhibited menstrual regularity and 18 female patients did not exhibit galactorrhea. Prior to surgery, 2 male patients exhibited sterility and decreased sexual function. Half a month post-EETA, such patients exhibited normal sexual function. Visual function postsurgery was also markedly attenuated, with 15 patients (28.8%) exhibiting an improvement and even complete normalization of visual function, and all 18 patients exhibiting a normalization of visual field defects postsurgery. The remaining patients who had exhibited normal vision function prior to surgery maintained normal vision postoperatively. All the 22 patients who had exhibited preoperative headaches demonstrated an attenuation of such symptoms. Seven (13.5%) patients had been treated with bromocriptine prior to surgery, and also demonstrated beneficial postoperative effects. Six patients (11.5%) experienced a recurrence of giant adenomas, invasion, and increased PRL levels following surgery.

Postoperative PRL levels in 36 patients were revealed to be markedly attenuated at the 1 and 7 days time intervals postsurgery, and 26 patients (50%) exhibited healthy serum levels (6.1–17.49 ng/mL) following surgery. However, 8 patients (15.4%) either demonstrated no change in PRL levels postsurgery or demonstrated an increase (30.79∼>200 ng/mL) in PRL levels postsurgery. The remaining 10 patients (19.2%) exhibited a PRL level of <75% compared with preoperative PRL levels. Furthermore, 1 out of 7 patients who took bromocriptine before surgery demonstrated a lower PRL level before taking bromocriptine, and there were no changes in the PRL levels for the other 6 patients.

Univariate analysis demonstrated that the defect of postoperative recovery was associated with tumor size, preoperative PRL levels, and invasion of prolactinomas (Tables 6–8). The results revealed that preoperative treatment with bromocriptine as well as varying nationalities did not affect the success rate of surgery (Tables 9 and 10). Logistic regression analysis revealed that only tumor size [*P* = .007; OR (95% CI) = 5.748 (1.621–20.379)] and preoperative PRL levels [*P* = .006; OR (95% CI) = 3.866 (1.464–10.212)] were associated with unsatisfactory postoperative outcomes (Table 11).

**Table 6:**
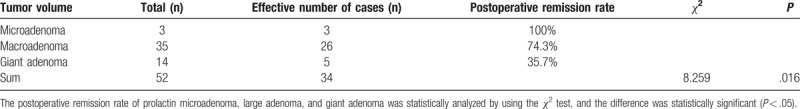
The relationship between the size of prolactin adenoma and postoperative relief.

**Table 7:**
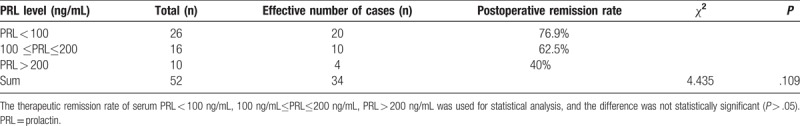
The relationship between preoperative PRL level and postoperative relief.

**Table 8:**
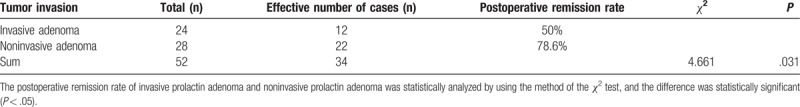
The relationship between the invasion of prolactin adenoma and postoperative relief.

**Table 9:**
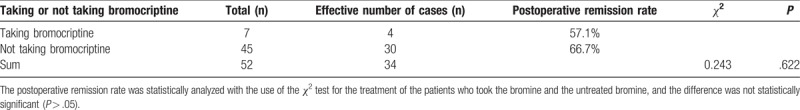
The relationship between taking or not taking bromocriptine and postoperative relief.

**Table 10:**
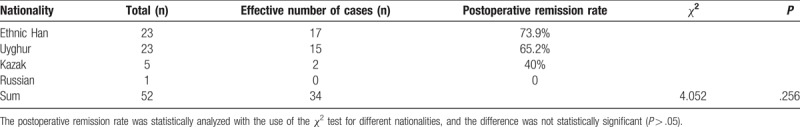
The relationship between different nationalities and postoperative relief.

**Table 11:**
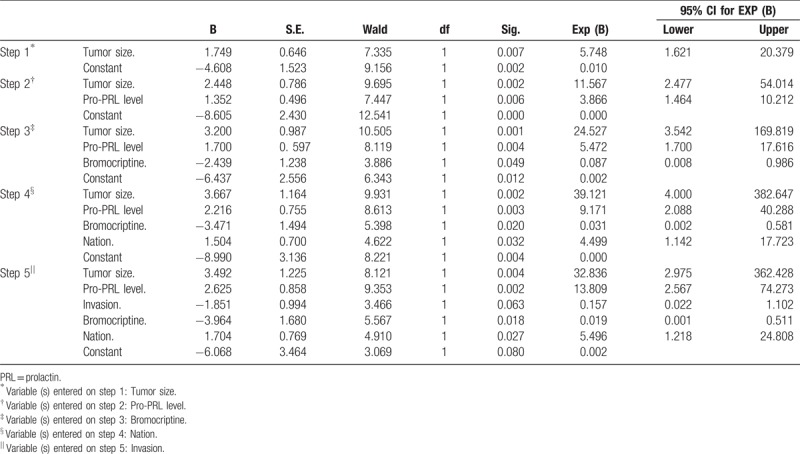
Variables in the equation.

### 3.4 Complications

A number of postoperative complications were exhibited by patients in the present study, including 2 patients (3.8%) with postoperative transient cerebrospinal fluid leakage and 5 patients (9.6%) with transient diabetes insipidus (TDI). Patients with cerebrospinal fluid leakage were successfully treated via endoscopic exploration and repair, and patients with TDI were successfully treated with desmopressin acetate. Meningitis, panhypopituitarism, apoplexy, and mortality were not observed in any of the included patients in the follow-up period.

## 4 Discussion

Although the preferred choice of therapy for patients with prolactinomas is treatment with DA, the majority of patients are finally treated via surgery. Surgical treatment is performed due to patients exhibiting drug resistance, drug intolerance, and drug-associated complications, such as pituitary haemorrhaging, visual disturbances, and giant tumors. In previous years, endonasal transsphenoidal surgery has been widely performed for the treatment of pituitary adenomas. Numerous studies have demonstrated that endoscopic transsphenoidal surgery for the treatment of pituitary adenomas is safer and more effective than the traditional microscopic approach. Endoscopic endonasal transsphenoidal surgery provides a panoramic visual field inside the surgical area, a superior view of the anatomy, an improved working angle, as well as reduced nasal cavity injuries.^[11]^ D’Haens et al^[12]^ demonstrated the endocrinological outcomes of patients subjected to these 2 different techniques; the overall remission rate of hypersecretion was 63% in the endoscopic group compared with 50% in the microsurgical group; the remission rate of hypersecretion investigated via endoscopy was 78% compared with 43% using microsurgery in patients with noninvasive macroadenomas; both endoscopy and microsurgery analyses revealed similar endocrinological results for patients with microadenomas; and a greater number of patients with postoperative cerebrospinal fluid leakage (6 patients) were identified via endoscopy compared with microsurgery. Akin et al^[13]^ investigated the tumor characteristics and clinical outcomes of 142 patients with prolactinomas treated using EETA via retrospective analysis of clinical data, which represents the first large study of patients with prolactin adenomas treated solely using an endoscopic approach. The results demonstrated that 45.8% of patients experienced remission shortly following surgery, and at the termination of the follow-up period, 74.6% of patients experienced remission following additional medical treatment. Raverot et al^[14]^ investigated 94 patients with prolactinoma treated by endoscopic transsphenoidal surgery and demonstrated that persistently elevated PRL levels were associated with increasing age, male sex, high preoperative PRL levels, and large tumor volumes according to univariate analysis. The results of the present study demonstrated that high preoperative PRL levels, large tumor volumes, and invasive prolactinomas were associated with persistent disease, in multivariate analysis, and influenced postoperative improvement.

Stalk effect, pituitary stalk compression syndrome, or disconnection hyperprolactinemia occurs when a pituitary tumor disrupts dopamine release by compressing the pituitary stalk and may, therefore, be accompanied by modest hyperprolactinemia.^[15]^ Nonsecretory pituitary macroadenoma causing hyperprolactinemia is attributed to the stalk effect. It is sometimes clinically challenging to differentiate a prolactin-secreting tumor from disconnection hyperprolactinemia because some cases demonstrate high-serum prolactin levels. But there were no statistically significant correlations between the pre- or postoperative pituitary volumes and increased serum prolactin levels for disconnection hyperprolactinemia. In this study, 52 patients were all pathologically diagnosed as prolactin-secreting pituitary adenomas. The result shows there was no stalk effect in 52 patients. The introduction of the 2-site monoclonal “sandwich” assays, immunor-adiometric assay (IRMA) and chemiluminometric assay (ICMA) (chemiluminescent assays) has been associated with more rapid and accurate hormone measurement.^[16]^ In these assays, 2 different antibodies, one attached to a solid surface (capture antibody) and the other added with patient's serum sample (signal antibody) label the hormone (antigen) with a substance such as radioactive tracer. After the unbound signal antibodies are washed away, the remaining tracer detected correlates to the “level” of prolactin hormone. However, if antigen out numbers capture and signal antibodies, the excess soluble antigen binds separately to each antibody. This prevents the formation of a “sandwich,” which leads to much lower levels of antigen detected. This is called high dose “hook effect” or “prozone phenomenon.” ^[17]^ The outcomes of 52 patients’ PRL levels were all accurate data from the clinical lab. The PRL levels were lower than most prolactinomas for some patients with macroadenomas and large adenomas. There may be a hook effect in those patients.

In the present study, 32 patients exhibited total resection of prolactinoma tumors and 20 patients exhibited a subtotal resection of prolactinoma tumors following surgery. The total removal rate of the 3 patients with microadenomas was 100%; the total removal rate of the 35 patients with macroadenomas was 68.6% and the total removal rate of the 14 patients with giant adenomas was 35.7%. Furthermore, the 24 patients with invasive prolactin adenomas exhibited a total removal rate of 29.2%, and the 28 patients with noninvasive prolactin adenomas exhibited a total removal rate of 89.3%. Statistical analysis revealed that the total excision rate of prolactin adenomas was closely associated with tumor volume and invasion. The results of the present study revealed that 34 patients exhibited improved clinical outcomes postsurgery, and the remission rate was 65.4%. Six patients exhibited tumor recurrence and a further 2 patients were rehospitalized during the follow-up period. The majority of patients suffered from macroadenomas, giant adenomas, and invasive prolactin adenomas, which were revealed to affect postoperative remission rate. Therefore, the postoperative remission rate was demonstrated to be low in the present study. The remission rates of patients with microadenomas, macroadenomas, and giant adenomas were 100%, 74.3%, and 35.7%, respectively. Considering that only 3 patients with microadenomas were included in the present study, the small sample size cannot be considered to be representative of statistically significant differences. However, the numbers of patients with macroadenomas and giant adenomas in the present study were large. Compared with patients with giant adenomas, the remission rate of patients with macroadenomas was increased. Therefore, the results of the present study suggested that endoscopic endonasal transsphenoidal surgery has a greater therapeutic effect in patients with macroadenoma tumors, compared with patients with giant adenoma tumors. The remission rates of noninvasive and invasive prolactin adenomas were revealed to be 78.6% and 50%, respectively, and the difference in the remission rates between the 2 adenoma types was demonstrated to be statistically significant (*P* < .05). Furthermore, postoperative relief was revealed to not be significantly associated with preoperative PRL levels (Table 7). The results demonstrated in the present study are in contrast with other studies, which may be due to the small sample size. In the PRL <100 ng/mL group, there were a large number of giant adenomas and invasive prolactin adenomas, which affected the postoperative remission rate. Furthermore, in the PRL 100 to 200 ng/mL group, numerous patients exhibited macroadenomas and 5 patients exhibited giant adenomas, which was also revealed to have a significant effect on postoperative remission rate. Therefore, it can be suggested that postoperative relief is associated with preoperative PRL levels. Seven patients included in the present study were administered bromocriptine prior to surgical intervention. In addition, the postoperative remission rate of patients who were administered bromocriptine was 55.1%, and the postoperative remission rate of patients who were not administered bromocriptine was 66.7% (Table 9); however, this difference was not statistically significant. Therefore, the results suggested that preoperative administration of bromocriptine does not have a significant effect on the therapeutic outcome of endoscopic endonasal transsphenoidal surgery. In addition, the present study included 23 patients of Han nationality, 23 patients of Uygur nationality, 5 patients of Kazak nationality, and 1 patient of Russian nationality. The results demonstrated that the postoperative remission rate of Han patients was 73.9% and the postoperative remission rate of Uygur patients was 65.2% (Table 10); however, this result was not statistically significant. Considering that the number of patients of Kazak and Russian nationality was small, any differences were not considered to represent statistically significant differences. Therefore, it can be concluded that the effect of surgical treatment on prolactin adenoma has no association with differences in nationality.

Giant prolactinomas are less than microadenoma and macroadenoma. At present, there is no consensus regarding the definition of giant prolactinomas. In the present study, an adenoma exhibiting a diameter of > 3 cm was considered to represent giant adenomas. Various therapeutic strategies, such as DA monotherapy or a combinatory treatment of radiotherapy and surgery, may be necessary to achieve desired therapeutic outcomes in patients with giant prolactinomas.^[18]^ Maiter and Delgrange^[19]^ suggested that a specialized multidisciplinary approach is necessary for the treatment of patients with giant prolactinomas. Fourteen patients with giant tumors were treated via EETA in the present study. In recent years, the efficiency of EETA in the removal of giant adenomas has been greatly recognized.^[20–24]^

Berker et al^[7]^ revealed that in 570 patients with pituitary adenoma that had previously undergone EETA, the incidence of complications following the surgery was minimum, and thus EETA could be considered safe as the microscopic transsphenoidal approach. EETA has marked advantages compared with traditional therapy, such as improved surgical visualization, less damages, decreased duration of time spent in hospital, and increased quality of life for patients exhibiting total resection postsurgery. In the present study of 52 patients with prolactinomas, 2 patients (3.8%) suffered from postoperative transient cerebrospinal fluid leakage and 5 patients (9.6%) suffered from TDI.

At present, surgical therapy of patients with prolactinomas remains controversial, and no consensus has been reached as to whether surgical treatment should represent the first line of therapy for patients with prolactinomas. Furthermore, EETA is necessary for patients who are intolerant or nonresponsive to Das treatment. Surgery may represent the preferred treatment strategy for patients exhibiting acute complications, such as apoplexy and cerebrospinal fluid leakage.^[25]^ Couldwell et al^[4]^ suggested that whether or not pituitary surgery represents the most effective therapeutic strategy for the treatment of patients with prolactinomas. It should be determined based upon the volume and location of the adenoma, the age of the patient as well as the efficacy and tolerability of the patient to DA treatment. Frank et al ^[26]^ demonstrated that the overall rate of biochemical remission of 66 patients with prolactinomas following endoscopic surgery was 76%. In addition, Dehdashti et al ^[27]^ investigated 25 patients and revealed that 96% of patients with microadenomas and noninvasive macroadenomas underwent endocrinological remission. Yano et al ^[28]^ published similar results in 2009, revealing that 94% of patients with microadenomas and 42% of patients with macroadenomas exhibited endocrinological remission. Considering this, it can be suggested that EETA may represent a preferred therapeutic strategy for the treatment of patients with prolactinomas whenever patients are not responding to medical therapy, or clinical symptoms are rather obvious and affected quality of life.

Previous studies have suggested that patients with prolactinomas may only exhibit a response 3 to 6 months following medical treatment alone. Furthermore, these patients with prolactinomas may exhibit a more rapid improvement in visual deficits. Therefore, the results of the present study suggested that surgery should be performed in patients who refuse prolonged medical treatment or who require short-term therapeutic outcomes. In this study, 1 out of 7 patients who took bromocriptine exhibited lower postoperative PRL but there were no improvements in visual loss after 3 months. Other 6 patients exhibited no changes in postoperative PRL and visual loss. The tumor size did not reduce after taking bromocriptine for 7 patients. At this point, we thought medical therapy has failed. So, these 7 patients underwent EETA, and surgical outcomes were well.

However, whether a patient should be subjected to surgery during medical treatment remains contentious. Patients who are nonresponsive to DA therapy should be considered for surgical treatment. Molitch^[8]^ defined DA resistance as failure to achieve normal PRL levels or as failure to achieve a reduction in tumor size of at least 50% following treatment with maximal conventional doses of medication. Kupersmith et al^[29]^ defined bromocriptine resistance as reduced prolactin levels despite a 15 to 30 mg daily dose of bromocriptine during a minimum of a 6-month treatment period. In addition, it has been well established that DA induces peritumoral fibrosis when administered for prolonged periods of > 3 months.^[29–32]^ Therefore, the results of the present study suggested that the ideal time period for determining whether a patient is suitable for endoscopic endonasal transsphenoidal surgery is once it has been established that the patient is resistant to DA treatment, but is not exhibiting substantial fibrosis prior to surgery.

However, there were several limitations in the present study. A total of 48 patients with macroadenomas or giant adenomas (92.3%) accounted for the majority of patients included in the present study. This may have affected the outcome of postoperative effects, and thus the expected effects cannot be obtained. Other endocrine abnormalities were at normal levels and did not correlate with postoperative result for 52 patients with prolactinomas. In addition, a control group of patients treated with solely microscopic transsphenoidal surgery was not included in the present study. It has been well established that EETA is a safer, more convenient, and efficacious therapeutic strategy for the resection of pituitary adenomas, and thus there was no requirement to perform microscopic surgery for patients with prolactinomas in the present study.

## 5 Conclusion

The results of the present study demonstrated that tumor size, preoperative PRL levels, and invasion of adenomas are independent factors that affect the therapeutic outcomes of patient postsurgery. In addition, the results suggested that tumors should be resected to the greatest degree possible, and that serum PRL levels of patients require monitoring for extended durations of time postsurgery to improve the remission rate and quality of life of patients. Finally, EETA may represent a primary treatment for patients with prolactinomas who do not respond to medical therapy.

## Author contributions

**Conceptualization:** Yong-Gang Wu.

**Data curation:** Yan-Long Han, Cheng Zhang.

**Formal analysis:** Yong-Gang Wu, Piao Pan.

**Investigation:** Dongming Chen, Cheng Zhang.

**Methodology:** Yan-Long Han, Dongming Chen.

**Project administration:** Yong-Gang Wu, Xiaopeng Yang.

**Resources:** Yan-Long Han, Xiaopeng Yang.

**Software:** Yan-Long Han, Dongming Chen, Cheng Zhang, Xiaopeng Yang, Piao Pan.

**Supervision:** Xiaopeng Yang.

**Validation:** Dongming Chen.

**Writing – original draft:** Dongming Chen.

**Writing – review & editing:** Yong-Gang Wu.
